# Extracellular histone release by renal cells after warm and cold ischemic kidney injury: Studies in an *ex-vivo* porcine kidney perfusion model

**DOI:** 10.1371/journal.pone.0279944

**Published:** 2023-01-20

**Authors:** Tim C. van Smaalen, Danielle M. H. Beurskens, Jasper J. H. F. M. Kox, Rasheendra Polonia, Rein Vos, Hans Duimel, Willine J. van de Wetering, Carmen López-Iglesias, Chris P. Reutelingsperger, L. W. Ernest van Heurn, Carine J. Peutz-Kootstra, Gerry A. F. Nicolaes

**Affiliations:** 1 Department of Surgery, Maastricht University Medical Center, Maastricht, The Netherlands; 2 Department of Biochemistry, Cardiovascular Research Institute Maastricht (CARIM), Maastricht University, Maastricht, The Netherlands; 3 Department of Methodology and Statistics, School for Public Health and Primary Care (CAPHRI), Faculty of Health, Medicine and Life Sciences, Maastricht University, Maastricht, The Netherlands; 4 Microscopy CORE Lab, Maastricht Multimodal Molecular Imaging Institute, FHML, Maastricht University, Maastricht, The Netherlands; 5 Department of Pathology, Maastricht University Medical Center, Cardiovascular Research Institute Maastricht (CARIM), Maastricht, The Netherlands; Khalifa University of Science and Technology, UNITED ARAB EMIRATES

## Abstract

Extracellular histones are cytotoxic molecules involved in experimental acute kidney injury. In patients receiving a renal transplant from donors after circulatory death, who suffer from additional warm ischemia, worse graft outcome is associated with higher machine perfusate extracellular histone H3 concentrations. We now investigated temperature-dependent extracellular histone release in an *ex vivo* porcine renal perfusion model, and subsequently studied histone release in the absence and presence of non-anticoagulant heparin. Seven pairs of ischemically damaged porcine kidneys were machine perfused at 4°C (cold ischemia) or 28°C (warm ischemia). Perfusate histone H3 concentration was higher after warm as compared to cold ischemia (median (IQR) = 0.48 (0.20–0.83) μg/mL vs. 0.02 (0.00–0.06) μg/mL; p = .045, respectively). Employing immune-electron microscopy (EM), histone containing cytoplasmic protrusions of tubular and endothelial cells were found after warm ischemic injury. Furthermore, abundant histone localization was detected in debris surrounding severely damaged glomerular cells, in a “buck shot” pattern. *In vitro*, histones were cytotoxic to endothelial and kidney epithelial cells in a temperature-dependent manner. In a separate *ex vivo* experiment, addition of heparin did not change the total histone H3 levels observed in the perfusate but revealed a continuous increase in the level of a lower molecular weight histone H3 variant. Our findings show that ischemically damaged kidneys release more extracellular histones in warm ischemia, which by EM was due to histone release by renal cells. Blocking of histone-mediated damage during transplantation may be beneficial in prevention of renal injury.

## Introduction

Histones are nuclear proteins, and the core histones H2A, H2B, H3 and H4 organize DNA into chromatin and regulate gene expression [1, 2]. The pathologic release of extracellular histones is known to have clinical consequences as these proteins are cytotoxic towards host tissues [3–6] and serve as damage-associated molecular patterns (DAMPs) [7]. Histone release can occur through regulated or unregulated cell death [8, 9] or through active immune cell-mediated expulsion [10, 11]. The presence of extracellular histones is linked to the initiation and detrimental progression of several clinical conditions that include organ specific events like acute kidney injury (AKI), acute lung injury (ALI) but also other conditions like sepsis, COVID-19, systemic lupus erythematosous (SLE), rheumatoid arthritis (RA), stroke and myocardial infarction [12–19]. The detrimental effects of extracellular histones have thus been studied in a variety of situations, including in renal disease. Extracellular histones have been associated with ischemia-reperfusion injury (IRI) in kidneys [5, 20, 21]. In IRI, an ischemic insult is followed by reperfusion with activated leukocytes, implying that circulating histones can be derived from intrarenal leucocytes and/or renal cells. Extracellular histones function as DAMPs that can activate cells through TLR2 and TLR4 receptors in post-ischemic and septic acute kidney injury and may aggravate kidney injury [20]. Direct injection of histones into murine kidneys results in increased levels of neutrophil recruitment, tubular injury and renal failure, effects that are abrogated when anti-histone antibodies are administered [20]. Moreover, histones play an important role in the adaptive immune response after kidney translation. Histone modifications contribute to the control of expression of major histocompatibility complex (MHC) I and II [22]. Serum concentrations of histone H3, as marker of circulating cell-free nucleosomes, were shown to be significantly higher in patient with acute rejection [23].

Acute kidney injury (AKI) is associated with high morbidity and mortality and is most often caused by cardiovascular or septic shock [24]. AKI also occurs after kidney transplantation, and is more severe after transplantation of kidneys from donation after circulatory death (DCD) donors, compared to kidneys from brain death (DBD) or living donors [25]. DCD kidneys suffer from additional warm ischemia in the donor, i.e. between circulatory arrest and organ preservation, leading to delayed graft function and longer hospitalisation [25]. There is a persisting donor organ shortage and a strategy to increase the donor pool may be to improve the organ quality of DCD donors [26], and thereby reduce rejection and adverse effects in the receiver. We recently found that high histone H3 concentrations in machine perfused kidneys prior to transplantation are associated with worse transplant outcome for DCD kidneys [27]. However, more knowledge on histone release from kidneys during transplantation, and on effects of histones on kidney viability, is needed to develop strategies aimed at preventing detrimental histone effects during kidney transplantation. *In vitro* studies have shown that dying tubular renal cells can release histones [20], and we hypothesize that ischemic renal tissue injury itself plays an important role in the histone release process. We specifically aim to study differences in temperature related severity of ischemic injury.

There is a general interest in the use of glycosaminoglycans to inhibit the cytotoxicity of extracellular histones and heparin is able to neutralize histones and thereby it may prevent histone accumulation on the endothelial glycocalyx and reduce histone cytotoxicity [13, 28, 29]. It has recently been shown that a high molecular weight heparin reduces ischemia reperfusion injury in machine perfused porcine kidneys [30]. Our experience with an antithrombin affinity-depleted heparin, that was shown to neutralize histone-mediated cytotoxicity in vitro and in vivo, in mouse models of inflammation and sepsis [13], led to the hypothesis that we could likewise neutralize histones during machine perfusion of kidneys.

To investigate histone release from ischemically injured kidneys in a transplantation setting, we used an *ex vivo* machine perfusion model [31]. The model allows the study of the relationship of extracellular histones with severity of injury during renal ischemia, in the absence of circulating immune cells. Furthermore, we studied temperature dependent kinetics of histone release by endothelial and renal tubular epithelial cells *in vitro*.

## Material and methods

### Study design

**Fig 1:**
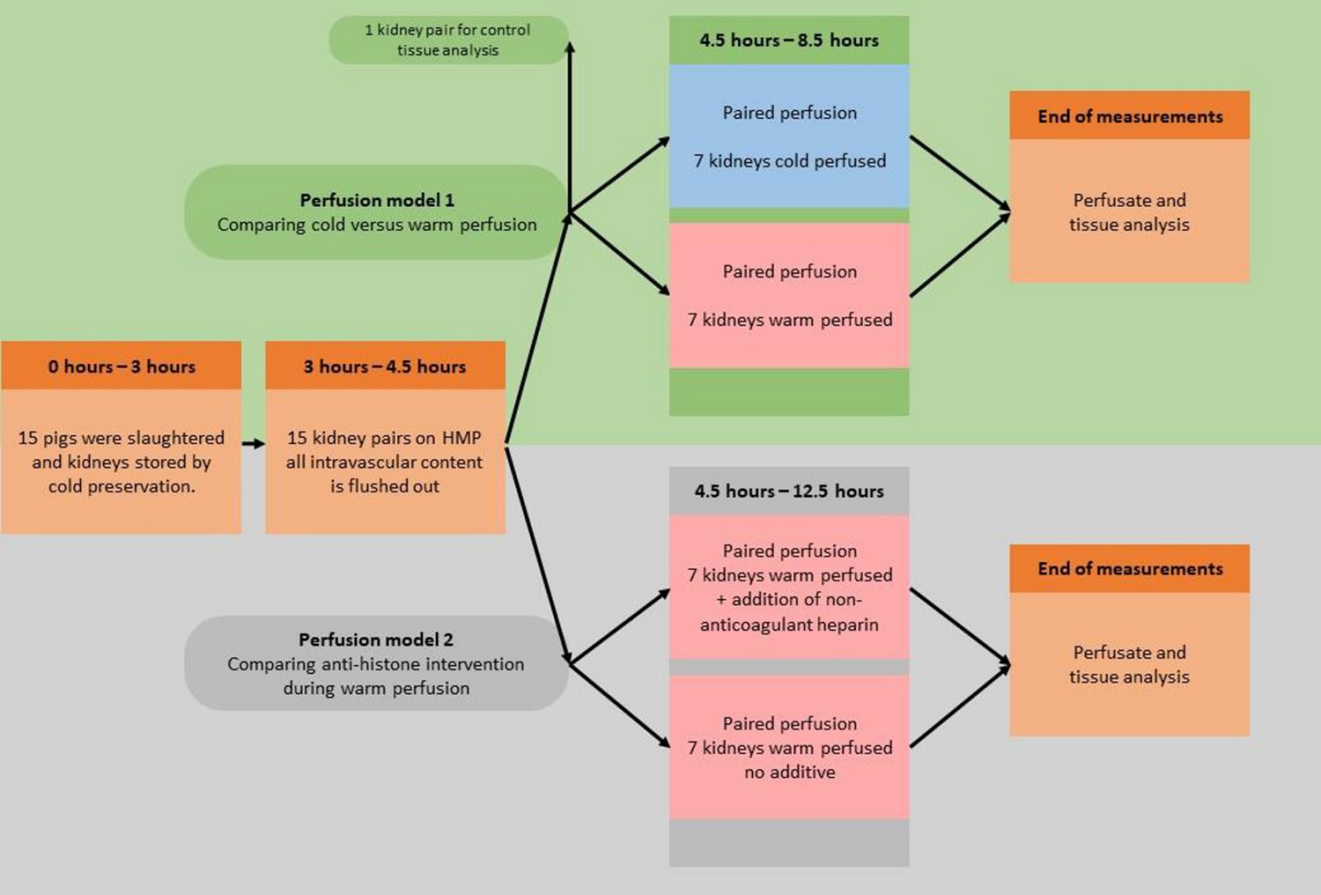
Paired ischemic porcine kidney perfusion models. Porcine kidneys are retrieved from the slaughterhouse and flushed 25min after sacrifice (warm ischemia time). Kidneys are then put on cold storage for 3h until the first period of cold machine perfusion at 4°C. Subsequently, kidneys are flushed one more time before being assigned to their group. **Model 1 (green)** includes 2 groups; 4h of cold perfusion vs 4h of warm perfusion. Regular perfusate sampling is done during this final perfusion period and at the end, biopsies are taken for further analysis. **Model 2 (gray)** includes 2 groups of warm perfusion; one kidney from a pair received non-anticoagulant heparin (0.31 mg/mL or 54 U/mL), whilst the other did not receive treatment. A total of 7 kidney pairs were used, from which 3 were perfused up to 4h and the other 4 pairs were perfused up to 8h. Regular perfusate sampling was done and tissue was collected at the end of perfusion.

In the porcine *ex vivo* perfusion models clinically relevant differences in warm vs cold ischemia were studied as described elsewhere [31]. In brief (Fig 1), 7 kidney pairs underwent ischemic kidney injury, while 1 kidney pair served as control. We retrieved all kidneys from a local slaughterhouse (Slachthuis Kerkrade) from Dutch Landrace pigs between 6 and 7 months of age and with a mean weight of 105 kg. After bilateral procurement, vasculature of both kidneys was flushed simultaneously with ice cold Histidine-Tryptophane-Ketoglutarate solution (HTK, Custodiol®). After cold storage, kidneys were simultaneously hypothermically perfused at 4°C using two Lifeport Kidney Transporters (model no. LKT-100-P; Organ Recovery Systems, Des Plaines, USA) for 1 hour with 0,5L of Kidney Perfusion Solution 1 (KPS-1; Organ Recovery Systems). After this first period of machine perfusion with two venous flush-outs, we assumed to have a pair of identical kidneys from which all intravascular content was removed. Thereafter, each kidney was assigned to either hypothermic (HMP) or subnormothermic (SNMP) machine perfusion (cold versus warm, respectively). HMP was performed similar to the initial perfusion period, SNMP comprised of kidney perfusion for 4 hours with KPS-1 at a temperature of 28°C. All kidneys were perfused at 40 mmHg, according to clinical standard practice. New perfusate and different perfusion packs were used for each machine perfusion period. Machine perfusate samples were taken from the Lifeport sampling port in the last 4 hour perfusion period and were immediately centrifuged at room temperature (RT) at 3000g for 3 minutes, and supernatants were aliquoted and stored at -80°C until further analysis.

In a second experiment, both kidneys were procured and handled in a similar manner as described above up to the final machine perfusion period (T4½, S1 Fig). Then, both kidneys from each pair were warm perfused for 4 hours (n = 7 pairs). One kidney from each pair was perfused for 4 hours with perfusion liquid containing 0.31mg/mL or 54 units/mL of nonanticoagulant heparin[13]. For 4 out of 7 kidney pairs, this was extended to 8 hours of warm perfusion time to study histone H3 levels over a longer time period.

### Perfusate analysis

Differences in metabolic activity between cold and warm perfused kidneys were analysed by measuring glucose (mmol/L), lactate (mmol/L), pH, pO_2_ (kPa) and pCO_2_ (kPa) with a blood gas analyser (GEM Premier 4000, Instrumentation Laboratory, Lexington, USA) using standardized laboratory methods. Lactate dehydrogenase (LDH, U/L) and total glutathione-*S*-transferase (GST, U/L) were measured using a Roche Cobas 8000 system (Roche Diagnostic International Ltd, Rotkreuz, Switzerland). Machine perfusion fluid samples taken at t = 10, 60, 120, 180 and 240 minutes were analysed. Perfusate taken at different time points was analysed for the presence of histone H3 using semi-quantitative Western blotting as previously described [18, 27].

### Renal biopsies and electron microscopy (EM)

For all kidneys, wedge biopsies were taken of renal parenchyma at the end of machine perfusion, except for the two kidneys that were used as controls, which were biopsied immediately after transport to our lab (Fig 1) which thus did not undergo any preservation.

For transmission EM, sections were chemically fixed at 4°C with a mixture of 4% paraformaldehyde and 0.1% glutaraldehyde in PHEM buffer (60mM PIPES, 25mM HEPES, 10 mM EGTA, and 4mM MgSO4*7H2O). After washing with PHEM containing 50 mM glycine, tissues were embedded in 12% gelatine and infused in 2.3 M sucrose. Mounted gelatine blocks were frozen in liquid nitrogen after which thin sections were prepared in an ultracryomicrotome (Leica EM Ultracut UC6/FC6, Vienna, Austria).

For immuno-EM, ultrathin cryosections were collected with 2% methylcellulose in 2.3 M sucrose. Then, cryosections were incubated at RT on drops of 2% gelatin/PBS for 20 minutes at 37°C, followed by 50 mM glycine/PBS for 15 minutes, 10% BSA/PBS for 10 minutes and 5% BSA/PBS for 5 minutes. Then they were incubated with anti-histone H3 antibody (1:50 in 5% BSA/PBS for 30 minutes at RT, ab1791, Abcam). After three washes of 10 minutes with drops of PBS, sections were incubated using protein A coupled to 10 nm diameter colloidal gold particles (1:50 in 1% BSA/PBS for 20 minutes at RT, AURION, Wageningen, the Netherlands). This was followed by another three PBS washes of 10 minutes and two washes of 10 minutes with distilled water. As a control for non-specific binding of the colloidal gold-conjugated antibody, the primary antibody was omitted. Observations were done in an Electron Microscope Tecnai G2 Spirit equipped with an Eagle 4kx4k CCD camera (FEI Company, The Netherlands).

### Cell culture experiments

The human endothelial cell line EA.hy926 (CRL-2922, ATCC) and porcine kidney proximal tubular epithelial cell line PK-15 (CCL-33, ATCC) were cultured in Dulbecco’s Modified Eagle Medium (DMEM) supplemented with 10% fetal bovine serum (FBS), 2 mM glutamine, 100 U/mL penicillin and 100 μg/mL streptomycin (all from Lonza, Walkersville, USA). In addition, EA.hy926 cells were supplemented with HAT: hypoxanthine (5 mM), aminopterin (20 μM) and thymidine (0.8 mM) (Lonza).

For *in vitro* cytotoxicity assays, the EA.hy926 and PK-15 cell lines were grown to confluence. Cells were washed twice with PBS before incubation with 5–40 μg/mL purified calf histone H3, 10 μg/mL purified calf histone H1 (both Roche, Sigma-Aldrich, St. Louis, MO, USA), 10 μg/mL human recombinant histones H2A, H2B and H4 (all New England Biolabs, Ipswich, USA) or 50 μg/mL histone mix (Roche, Sigma-Aldrich, St. Louis, MO, USA) for 1 hour in serum-free DMEM (Lonza, Switzerland) at 37°C in 5% CO2. Next, serum-free DMEM was collected in Low-Bind Eppendorf tubes and cells were washed once with PBS. Thereafter, cells were detached using 0.05% Trypsin-EDTA (0.05% Proteinase-K) for 5 min at 37°C in 5% CO2. Next, fresh DMEM (2mM glutamine, 100 unit/mL penicillin, 100 μg/mL Streptavidin, Lonza, Switzerland) was added to the cells and cells were collected by scraping. Cells were spun down at 600 g at 4°C and supernatant was removed. Cells were then solved in 10 mM HEPES, 150 mM NaCl, 5 mM KCl, 1 mM MgCl2 and 2.5 mM CaCl2 pH 7.4 buffer containing 0.25 μg/mL Annexin A5-FITC (PharmaTarget BV, Maastricht, The Netherlands) and 2.5 μg/mL propidium iodide (Invitrogen Life Technologies, Carlsbad, USA) and incubated for 10 minutes in the dark on ice. Cell viability was assessed by flow cytometry employing a BD Accuri C6 flow cytometer as previously described 19.

For immunofluorescence studies EA.hy926 cells were seeded and grown to confluence. Cells were equilibrated to 4°C, 22°C or 37°C before incubation with 10 μg/mL histone H3 in serum-free DMEM for 1 hour. Cells were then washed, stained with 2,5 μg/mL PI and 0,5 μg/mL Hoechst 33342 (ThermoFischer, Mannheim, Germany) for 10 minutes and imaged using a EVOS FL inverted digital microscope (ThermoFischer). Six random images were taken at 20x magnification per tested condition. The percentage of PI positive cells was quantified for each image using ImageJ software (x86, Wayne Rasband, National Institute of Health, USA) and then averaged to generate a mean PI positivity for each condition.

### Data analysis

All data were analysed using IBM SPSS statistics Version 22.0 (IBM Corp., Armonk, USA) or GraphPad Prism 5.0 (GraphPad Software Inc., La Jolla, USA). Numerical variables are presented as mean ± standard deviation (SD) if normally distributed or else as median (interquartile range (IQR)). Categorical variables are presented as numbers (percentage). For statistical analysis of immunohistochemical, flow cytometry and immunofluorescence data, one-Way ANOVA was performed, while applying a Bonferroni correction when comparing multiple groups and a Dunnett test when comparing groups to control. For analysis of histone concentrations in the perfusate, the non-parametric Friedman test was used to test if concentration changes recorded over time for machine perfusion groups (warm and cold) were significant and presented as Chi-square and p-value per group.

To describe the difference of concentration changes over time between both groups, we used a linear mixed model. This model was applied, assuming a linear regression model, in which both types of machine perfusion (warm and cold) and machine perfusion sample time were used as fixed effect and a random intercept was included. For determination of the difference between groups, we used the interaction term of these two parameters (type of machine perfusion * machine perfusion sample time) and presented the p-value. Extracellular histone release over time was investigated for each type of perfusion group. To compare concentrations of GST and glucose between machine perfusion groups (warm vs. cold), the Mann-Whitney test was used per point of machine perfusion sample time (t = 10, 60, 120, 180 and 240 minutes). The Friedman test was used to test if concentrations changed over time per aforementioned marker in both machine perfusion groups. In the intervention perfusion model, histone concentrations per time point were compared by performing an independent t-test. Significant differences were indicated by a *p-value of <0.05, **p-value of <0.01 or ***p-value of <0.001.

## Results

### Severity of warm versus cold ischemic renal injury in the machine perfusion model

The differences in ischemic renal injury between the models are shown by quantitation of several markers in the machine perfusate. In the machine perfusate, we determined uptake of glucose, a marker of metabolic activity, by kidneys after warm and cold ischemia over time (Friedman test: SNMP Chi-square 16.000, p = .003 and HMP Chi-square 18.000; p = .001; S1 Table). Glucose uptake was higher after warm ischemia. Furthermore, LDH, a widely used marker of tissue damage during machine perfusion [32], and lactate, as end product of renal metabolic activity, significantly increased over time, more so in warm perfusion compared to cold perfusion (S1 Table). The markers GST, pO_2_, pCO_2_ did not significantly differ between groups, while pH did. However, these markers are known to be unreliable markers of viability during machine perfusion (S1 Table). Immunohistochemical staining for MPO confirmed the absence of neutrophilic granulocytes in all perfused porcine kidneys, while an occasional neutrophil was found in control kidney sections.

### Histone detection in machine perfusate samples

**Fig 2:**
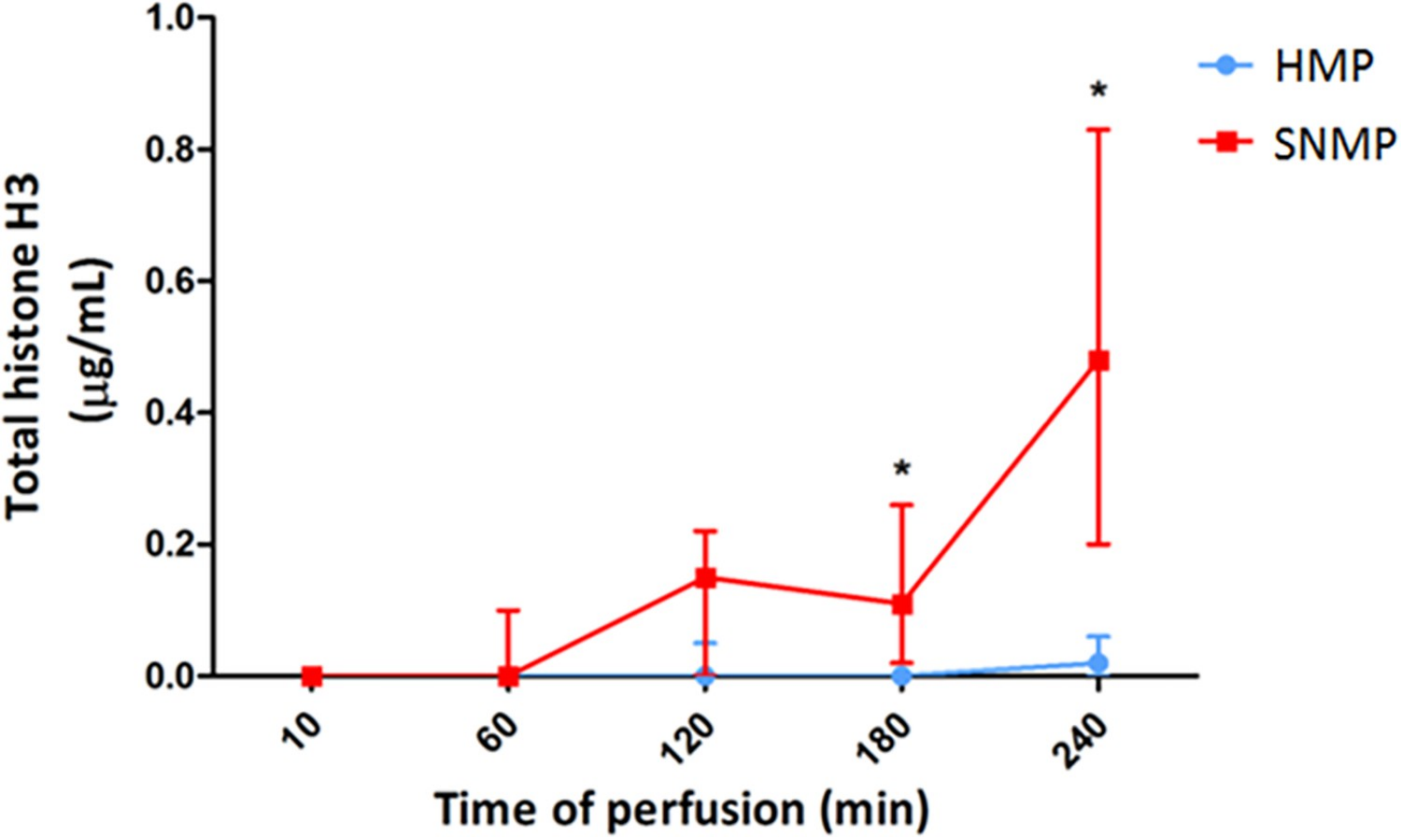
Release of extracellular histone H3 is dependent on the amount of ischemic injury. Extracellular histone H3 was determined semi-quantitatively using Western blotting. Perfusate levels of total histone H3 increased over time in the warm ischemic injury group, whilst remaining constant in the cold ischemia group. Data are presented as median and whiskers include interquartile range.*Indicates significant differences between groups (p<0.05).

Histone concentrations of the machine perfusate were measured to investigate ischemia dependent histone release into the extracellular space. During the initial stages (e.g. procurement, storage and initial flushing and perfusion stages) levels of extracellular histones in the perfusate were below the detection limit of the assay used (5 ng/ml histone H3 [33]. At the final stage of the machine perfusion, extracellular histones were detected (Fig 1). Histone H3 concentrations were significantly higher after 4 hours of warm versus cold ischemia (median concentration = 0.48 (0.20–0.83) μg/mL) vs. 0.02 (0.00–0.06) μg/mL; p = 0.045, respectively; Fig 2). Furthermore, there was a significant change in histone concentration over time in the warm ischemia group, while this was not the case in the cold ischemia group (Friedman test = χ^2^ = 44.559; p<0.001 vs. χ^2^ = 11.613; p = 0.393, respectively). This was also found using a mixed linear model (p-value for the estimate of fixed effect for the interaction term of machine perfusion group * time of machine perfusion p = 0.001).

### Electron microscopy (EM) studies of histone expression in ischemic renal injury

**Fig 3:**
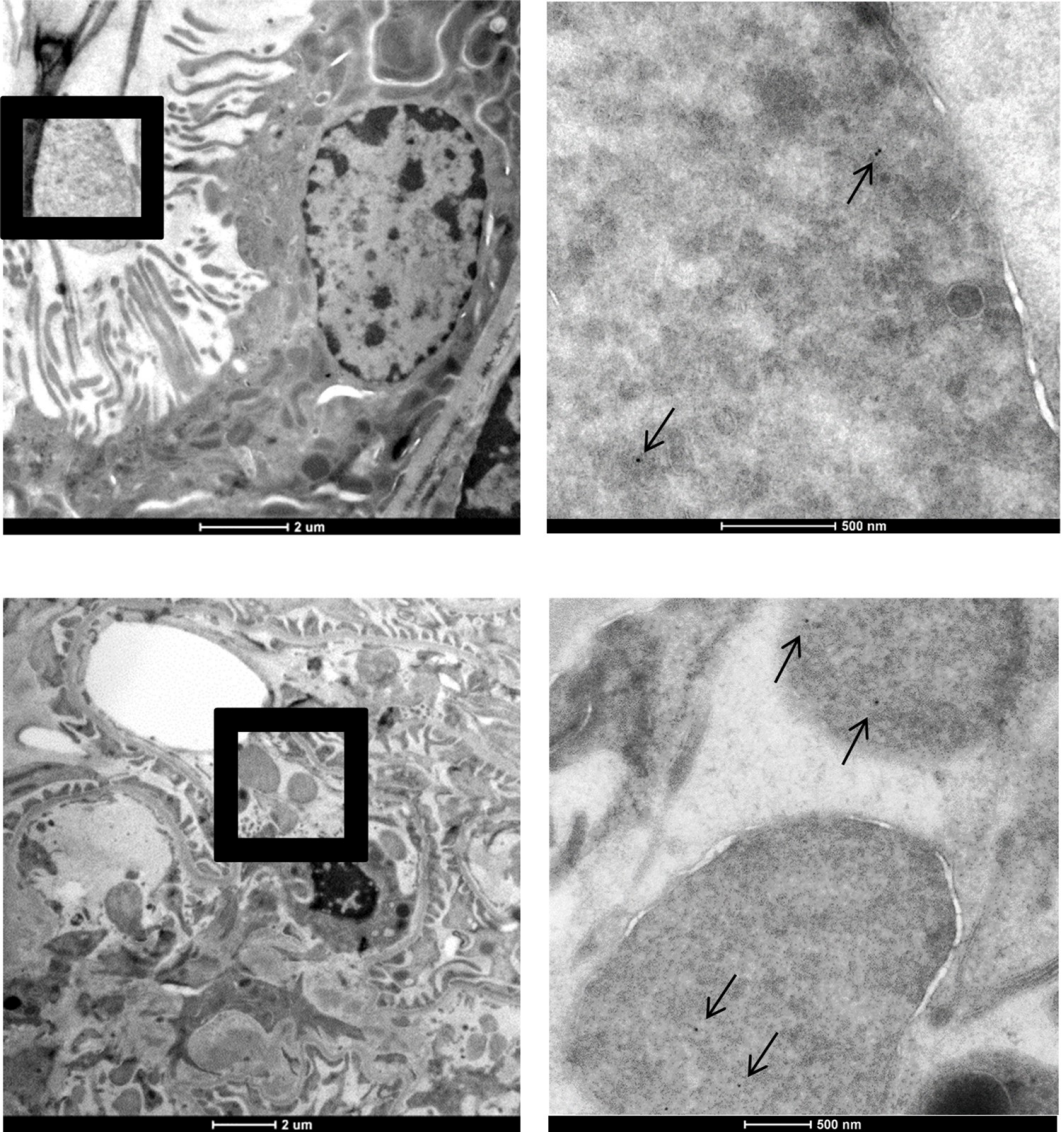
EM images of anti-histone H3 immunolabelling after 4 hours of warm ischemia in moderately damaged renal cells. Histone H3 (few gold particles indicated with black arrows) was scarcely detected by immuno-EM using antibody labelling with 10 nm gold particles. Top row: representative images are shown for histone H3 localization in a proximal tubule with histone localization in an ovoid cytoplasmic structure (left low magnification, right high magnification). Bottom row: extracellular histone H3 is present in ovoid and spheroid in glomerular endothelial cytoplasmic structures (left low magnification, right high magnification). Scale bar represents 2 μm or 500 nm as indicated.

**Fig 4:**
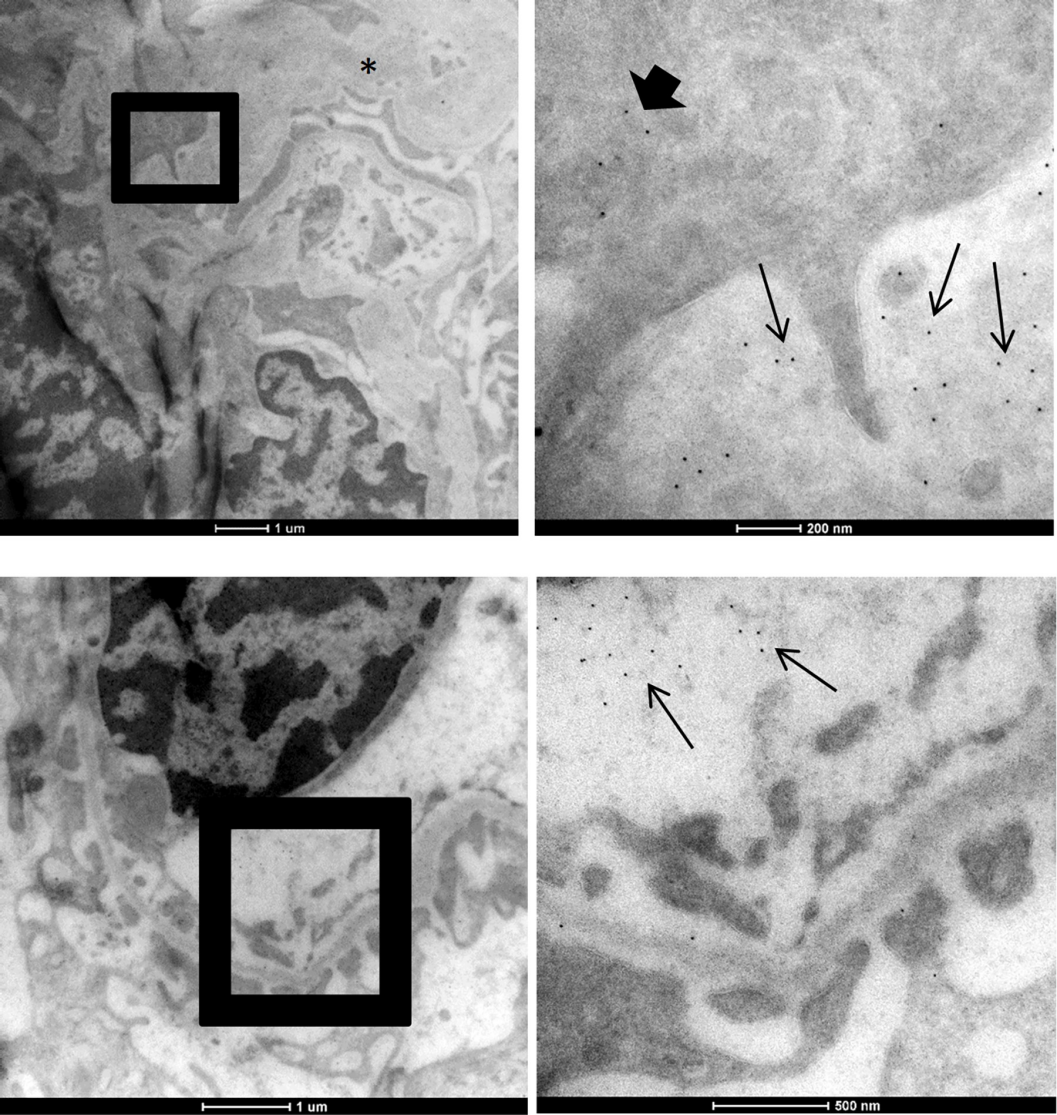
EM images of anti-histone H3 immunolabelling after 4 hours of warm ischemia in severely damaged glomerular cells. Histone H3 was detected by immuno-EM using antibody labelling with 10 nm gold particles (visible as small black dots). Top row: representative images are shown for histone H3 localization surrounding a podocyte with foot process effacement (asterisk, left image), with histones in the cytoplasm (thick arrow and in extracellular debris (thin arrows) in a “bug shot pattern”, right image). Bottom row: extracellular histone H3 is released from an endothelial cell with loss of endothelial fenestrations and subendothelial accumulation of electronlucent material (left image enlarged area in right image) with histone localisation in extracellular debris (thin arrows, right image). Scale bar represents 1 μm or 200 nm or 500 nm as indicated.

Transmission EM of kidney tissue after warm ischemia showed intraluminal spheroid or oval blebs protruding from tubular and endothelial cells in tubuli and peritubular and glomerular capillaries (S1 Fig), as described previously in ischemic renal injury [34, 35]. With immunogold electron microscopy, histone H3 was detected in nuclear chromatin of all cells, which thus served as internal positive control. After warm ischemia, histones H3 were additionally found in the cytoplasmic blebs protruding in lumina of glomerular and peritubular capillaries as well as in tubular lumina (Fig 3). Moreover, abundant localization of histones was detected in extracellular debris of severely damaged glomerular cells. Fig 4 shows a podocyte with extensive foot process effacement after warm ischemic injury with localization of histones by immuno-gold labelling in extracellular debris in a “buck shot pattern”. This pattern was also seen surrounding a mesangial cell. A similar pattern of histone expulsion was found surrounding endothelial cells with loss of fenestrations and subendothelial accumulation of electron-lucent material, while the adjacent podocyte still has intact foot processes.

### Cytotoxic effects of extracellular histones *in vitro*

Endothelial and renal epithelial cell viability decreases upon histone exposure in a concentration-dependent manner (S2 Fig). Endothelial cells incubated with different types of histones revealed a variable effect on cell viability for the different histone isotypes (S2 Fig). For these cells, histone-mediated cytotoxicity appears to be isotype specific, with the linker histone H1 being the least cytotoxic, followed by histone H2A, H2B, H3 and H4. The mixture of histones (consisting of all histone subtypes at equal molar ratio) induces the most cell death.

**Fig 5:**
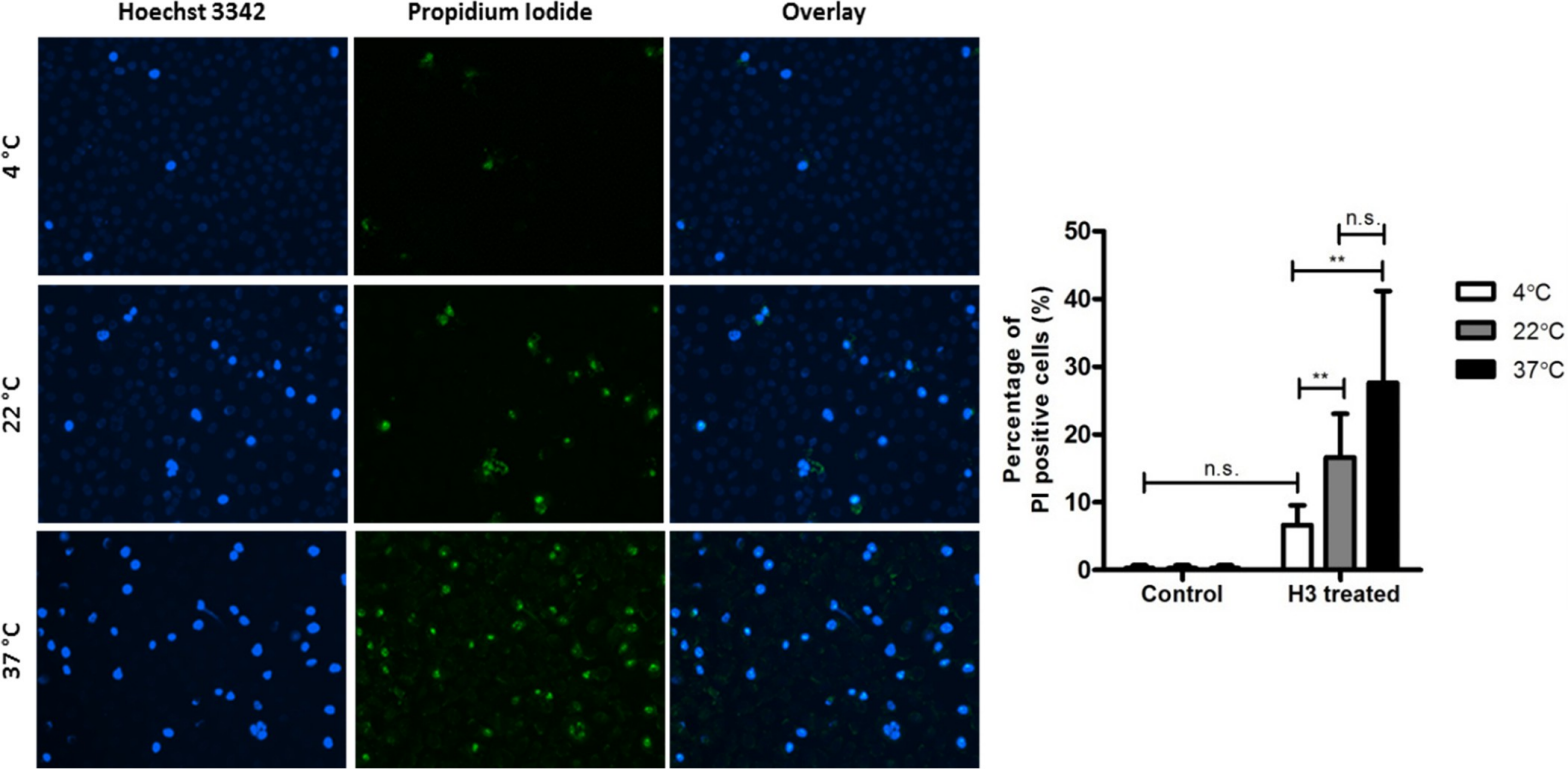
Temperature-dependent cytotoxicity of extracellular histone H3. Endothelial cells (EA.hy926) were incubated with 10 μg/mL histone H3 in serum-free DMEM for 1 hour at 4°C, 22°C or 37°C. Left panel: representative images from histone treated cells. Nuclei were stained using Hoechst 33342 (in blue) and necrotic cells were stained using PI (in green). Right panel: The number of PI positive cells were quantified in 6 random image fields using ImageJ software. Data is expressed as the mean ± SD of the average percentage of PI positive cells per condition (n = 5).

To investigate whether the cytotoxicity of extracellular histones is dependent on temperature, EA.hy926 cells were incubated with a fixed concentration of histone H3 for 1h at 4°C, 22°C or 37°C. Immunofluorescence analysis show that the number of PI positive nuclei, as a measure of histone-induced cell death, increases with increasing temperature (Fig 5), with incubation at 37°C resulting in significantly more cell death than incubation at 4°C (27.6%, p<0.01). Incubation at an intermediate temperature of 22°C induced a moderate level of cell death (16.6%) even though this was not significantly different from the levels of induced cell death for the other temperatures tested.

### Nonanticoagulant heparin and histone presence in kidney perfusates

**Fig 6:**
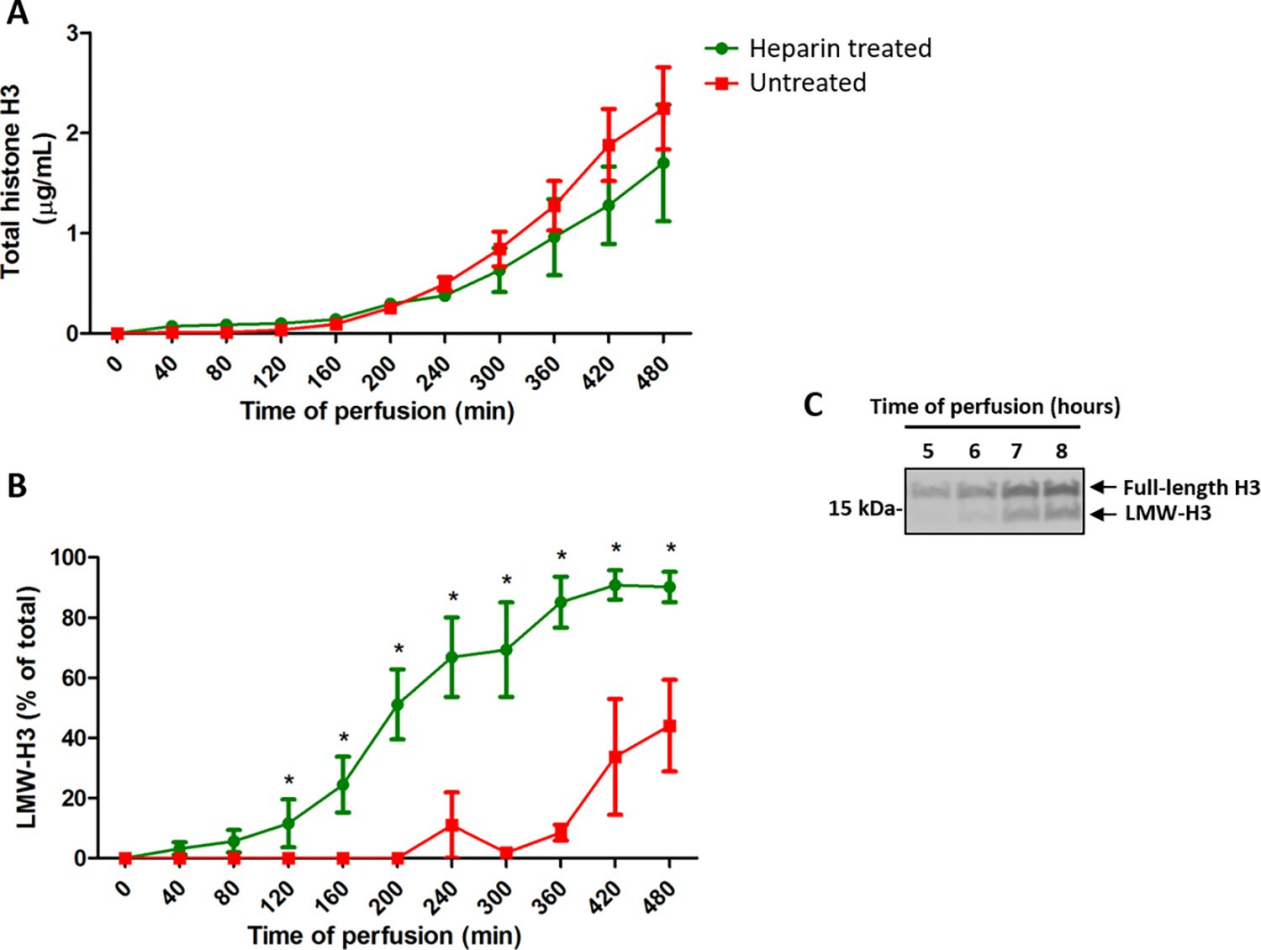
Time-dependent release of histone H3 in heparin treated and untreated kidneys with warm ischemic injury. Extracellular histone H3 was determined in kidneys with or without circulating nonanticoagulant heparin. Kidneys treated with heparin showed a similar release in total histone H3 (A) but a more rapid and pronounced increase in levels of a lower molecular weight histone H3 (LMW-H3) variant (B) compared to untreated counter kidneys. (C) A representative Western blot image of full-length histone H3 (17 kDa apparent MW) and LMW-H3 (15 kDa apparent MW) in perfusate of untreated SNMP kidney at t = 5, 6, 7 and 8 hours of perfusion. *Indicates significant differences between groups (p<0.05).

Addition of heparin at the start of warm perfusion did not result in changes in perfusion parameters and glucose perfusate levels as compared to kidneys that did not receive heparin. Western blot analysis of machine perfusate showed a continuous increase in the total amount of extracellular histone H3 that was detected in the perfusate up to the last measurement at 8 hours. However, no significant differences in the total amount of extracellular histone H3 between heparin treated or untreated kidneys at any time point up to 8 hours of perfusion were observed (Fig 6A). Interestingly, as was found by Western blotting, in both groups a histone H3 band of an apparent lower molecular weight of about ±14–15 kDa was found which gradually appeared over the course of perfusion time, clearly discernible from the full-length protein band with an apparent MW of ±17 kDa (UniProt F1RVA0 determined MW of 15.4 kDa) (Fig 6C).

While the total amount of histones that were measured in the perfusate was equivalent for both groups, the LMW-H3 was released significantly faster and more pronounced in kidneys with circulating heparin compared to their counter parts (Fig 6B). The increase in LMW-H3 became apparent for the heparin kidneys after 2 hours of perfusion whilst this only started to occur after 6 hours of perfusion for the untreated counterparts. For both groups, this increase was exponential up to at least 8h of perfusion. Significant differences in LMW-H3 levels between both groups were observed for all time points between 2 and 8 hours of perfusion.

## Discussion

Acute kidney injury can lead to delayed graft function and primary non function after kidney transplantation, especially in the context of DCD donors that suffer from extended warm ischemia in the donor [36, 37]. As high histone H3 concentrations in machine perfused kidneys prior to transplantation are associated with worse transplant outcome for DCD kidneys [27], we now investigated the pathophysiological role of histone release in ischemic kidney injury in an *ex vivo* porcine model with perfusion at 4°C (cold ischemia) and at 28°C (warm ischemia). We find that extracellular histones are released from ischemically damaged kidneys during machine perfusion into the perfusate in a time- and temperature-dependent manner. With EM immunolabelling, histones were located in cytoplasmic protrusions of tubular and endothelial cells of moderately damaged cells after warm ischemia. Furthermore, abundant histone localisation was found in extracellular debris surrounding severely damaged glomerular cells. In *in vitro* experiments we found that the cytotoxic effect of histones on renal cells depends on both temperature, histone concentration and histone isotype. While we have shown earlier [13] that nonanticoagulant heparin is able to neutralize histone mediated cytotoxicity towards endothelial cells *in vitro* and in an *in vivo* sepsis model, *ex vivo*, addition of nonanticoagulant heparin did not result in different overall histone H3 levels, but resulted in an earlier shift from a full length histone H3 release towards the appearance of a lower molecular weight histone H3 variant in the circulating perfusate. Our results show that histones can be released from ischemically injured renal cells, and that histones induce direct damage to renal cells in a temperature dependent manner. Thus, histone release can be an indicator of ongoing tissue damage, and actively contribute to warm ischemic kidney injury.

Extracellular histones were found in the machine preservation fluid of kidneys with substantial warm ischemic damage, whilst this release was not seen after cold ischemic injury. Previously, we found expression of lipid-degradation products in kidney tissue after warm but not cold ischemia with mass-spectrometry imaging in this model [31]. In the various experiments performed with the present in vivo model, we found higher levels of lactate and LDH and lower glucose levels in the preservation fluid as sign of increased renal injury. As we verified that the kidneys in our model were depleted of neutrophilic granulocytes due to the perfusion prior to ischemia, our findings indicate that the ischemically damaged renal cells themselves release histones. This in line with *in vitro* studies where dying tubular cells were capable of releasing histones [20]. While in our study we specifically looked at histone H3, we regard this isotype as a proxy for other isotypes as all core histone isotypes are complexed in nucleosomes and are expected to be released as such.

Histones have been detected by immunofluorescence in human biopsies of patients with post-transplant acute tubular necrosis and kidneys of mice with ischemia/reperfusion injury [5, 38]. In the human biopsies neutrophilic granulocytes were found in close proximity to the histones, indicative that the detected histones likely originate from neutrophil extracellular traps (NETs). Also in biopsies of patients with ANCA vasculitis, NETs have been detected adjacent to infiltrating neutrophils [39]. With EM immunolabelling we now find intracellular and extracellular histone H3 localization in glomerular and tubular cells of kidneys with warm ischemia, in a setting that is neutrophil-independent. In moderate warm ischemic injury, histones are localized in cytoplasmic blebs/protrusions of tubular cells [40] and endothelial cells in peritubular as well glomerular capillaries. Abundant extracellular localization of histones was found in severely damaged glomerular cells in a “buck shot” pattern within cellular debris. This pattern of histone expulsion resembles that of ETosis, as has been visualized in neutrophilic granulocytes (NETosis) [10] and macrophages (METosis) [40] with immuno EM. Given the absence of blood flow, and verified absence of neutrophils, our findings suggest that the histones are derived from renal cells and may form renal cell derived extracellular traps (RETs), parallel to NETosis or METosis pathways. These histones, may be equally harmful and can further aggravate renal injury by damaging adjacent cells in an autoamplification loop.

Our *in vitro* experiments confirm that extracellular histones exert cytotoxic effects on endothelial and tubular epithelial cells [20], which has also been found for glomerular endothelial cells and podocytes [41]. Cytotoxicity was observed most at 37°C and only minimal cell death at 4°C. Histone-mediated toxicity is known to progress through several pathways, including histone-mediated membrane disruption [17, 42], histone-mediated NOD2/NLRP3 activation [43, 44] and histone-induced TLR-activation [20, 45, 46]. Of relevance, it has been described that TLR receptors are upregulated in DCD kidneys [47]. Machine perfusion, one of the first preservation methods [48], is widely used as preservation method for donor organs [49, 50]. Hypothermic machine perfusion (HMP) shows superior transplant outcome compared to cold storage [51–53] and has the advantage that it reduces renal resistance during perfusion and flushes out vasoactive and inflammatory mediators, while oxygen and nutrients may be delivered [54, 55]. In contrast to HMP, normothermic machine perfusion (NMP) is recently gaining popularity, because it adds the advantage of perfusing organs while they are metabolically active and therefore injury may be prevented [56–58]. The clinical introduction of NMP [59, 60] makes our findings relevant for transplant immunology, because we show that histones are not only more cytotoxic at higher temperatures, but their presence is more pronounced after prolonged warm perfusion. Recent preclinical and clinical studies show the feasibility and safe use of normothermic perfusion up to 48 hours [61], which emphasizes the relevance of studying histone release during prolonged warm perfusion. Synchronously to the increase of normothermic perfusion, there is an increase of oxygenation during machine perfusion. In normothermic perfusion, oxygenation is necessary for graft viability. However, oxygenation during hypothermic machine perfusion is arbitrary. Many preclinical and clinical studies have been conducted, questioning the use of oxygen in a hypothermic environment [62]. While it should provide a benefit in theory, its clinical benefit is less clear. The only clinical publication using oxygenation in DCD kidneys was recently published in the Lancet [63]. The primary outcome measure, eGFR at one 1 year, did not differ significantly between oxygenated and non-oxygenated groups. Oxygenation was not an option in our perfusion model, using kidneys which were deprived of all intravascular content, because the use of the oxygen carriers may interfere with histone measurements and lead to additional bias. Further studies should clarify the role of histone release in oxygenated machine perfused kidneys.

The increasing appearance of cytotoxic proteins in the preservation fluid led us to test charge-mediated neutralization of these toxic molecules with nonanticoagulant heparin, which has reported histone-neutralizing properties *in vitro* and has previously been shown to reduce histone-induced cell death in inflammation *in vivo* [13]. Unexpectedly, within the timeframe of this experiment, the total amount of detected extracellular histone H3 did not differ between both groups, nor did markers of cellular damage in the preservation fluid. Histones are known to bind cell surface proteoglycans [64], but also to plastic tubing. We cannot exclude that heparin could possibly lead to an apparent increase in the circulating histone pool as it can serve as a carrier molecule able to liberate cell-bound histones or stabilize histones that were released and retain them in the circulating perfusate. Interestingly, we observed a progressive appearance of a lower molecular weight histone H3 variant (LMW-H3) in warm perfused kidneys, with heparin accelerating this phenomenon. The presence of this variant is hypothesized to be the result of histone proteolysis and/or modification. Binding of histones to heparin could lead to an increased affinity of (circulating) protease(s) for histone substrate and initiate proteolysis faster than in untreated kidneys. In the literature, several examples of histone-cleaving proteases have been described: trypsin(-like) enzymes [65], granzyme A [66], cathepsins [67], and activated protein C (APC) (own observations and [68], plasma hyaluronan-binding protein[69], neutrophil elastase (NE) [19, 70], mast cell tryptase [71], plasmin [72]. In addition, many other proteins can react with histones in plasma, including proteinase inhibitors and MMP-2 which might be blocked by heparin binding [73]. Interestingly, MMP-2 expression is linked to delayed graft function after kidney transplantation [74] while cleavage of histones has been associated to reduced development of acute kidney injury in severe COVID-19 ICU patients [19]. Future studies are needed to clarify the exact origin and role of histone variants after addition of heparin, and cytotoxicity of the smaller histone variant, also in the context of ischemic kidney injury.

A limitation of this study is that our model did not allow the accurate measurement of functional markers such as creatinine, blood urea nitrogen, urinary markers and urine output [75, 76], as kidneys are deprived from blood and do not function as *in vivo*. Novel preservation strategies such as oxygenated NMP with autologous blood should be considered in order to better assess graft function [77]. A more direct translational approach would be the use of a porcine transplantation model. Finally, as we did not label our heparin, we could not investigate in more detail the localization of this polysaccharide in the machine perfused kidney. Sedigh et al. have shown that a biotinylated heparin conjugate bound to the inner surface of the vessel wall during machine perfusion of porcine kidneys and could restore the endothelial glycocalyx [78]. Although they did not observe this for unfractionated heparin, it remains unclear whether the unfractionated heparin could be visualized with fluorescently labelled streptavidin.

In conclusion, our findings substantiate the role of histones during warm ischemic kidney injury, and show that damaged renal cells themselves can release extracellular histones. This histone release can further aggravate ischemic renal injury. Translational studies should now be encouraged to investigate the clinical effect of histone release and histone neutralization, because of the increasing use of donor kidneys with increased warm ischemic injury and the exponential use of normothermic perfusion in the field of kidney transplantation.

## Supporting information

S1 Fig Transmission EM images after 4 hours of cold (left) or warm ischemia (right). In cases with warm ischemia, cytoplasmic blebs (arrows) are seen in lumina of proximal tubuli with cytoplasmic blebbing and swelling in peritubular capillaries (surrounded by square). Scale bar represents 5μm.
(TIF)

S2 Fig Cytotoxicity of extracellular histones to endothelial and kidney epithelial cells. (A) Endothelial cells (EA.hy926) and porcine kidney epithelial cells (PK-15) were incubated with 5–40 μg/mL histone H3 in serum-free DMEM for 1 hour at 37°C. (B) PK-15 cells were incubated with 10 μg/mL of individual histones H1, H2A, H2B, H3 and H4, or a mixture of these histones (Hmix) under the same conditions as in (A). Cell viability was assessed using flow cytometry with PI and annexin-A5 FITC. Data is shown as the mean + SD. *p<0.05 and ***p<0.001 as compared to control.
(TIF)

S1 Table Friedman and Mann-Whitney tests for differences between type of perfusion group per time point. (PDF)
