# Supplementary figures and images for: Extracellular histone release by renal cells after warm and cold ischemic kidney injury: Studies in an ex-vivo porcine kidney perfusion model
Source: PLoS One. 2023 Jan 20;18(1):e0279944. doi: 10.1371/journal.pone.0279944 (PMC9858092; doi:10.1371/journal.pone.0279944)

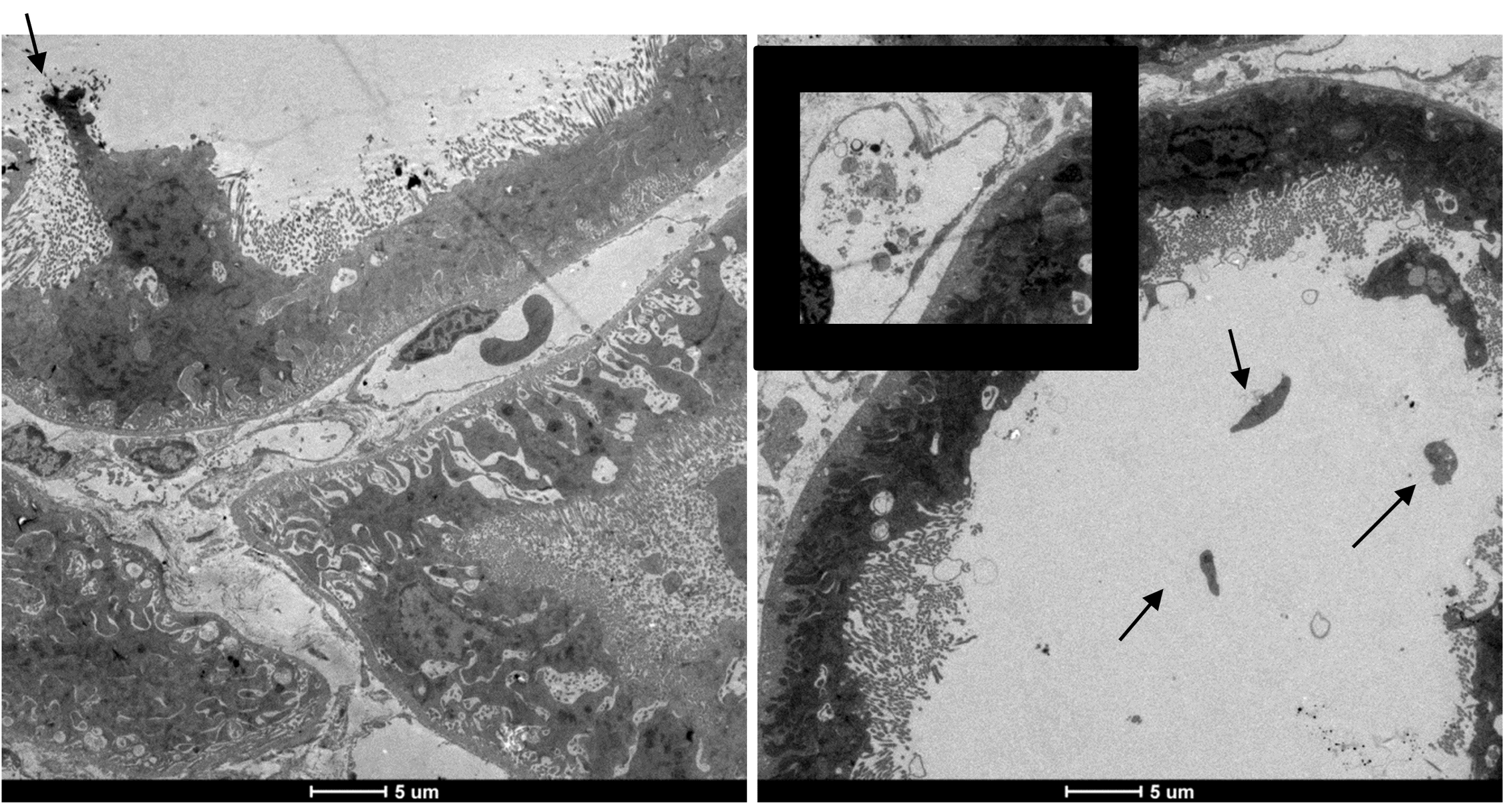

Supplement: S1 Fig — In cases with warm ischemia, cytoplasmic blebs (arrows) are seen in lumina of proximal tubuli with cytoplasmic blebbing and swelling in peritubular capillaries (surrounded by square). Scale bar represents 5μm. (TIF) [file pone.0279944.s001.tif]

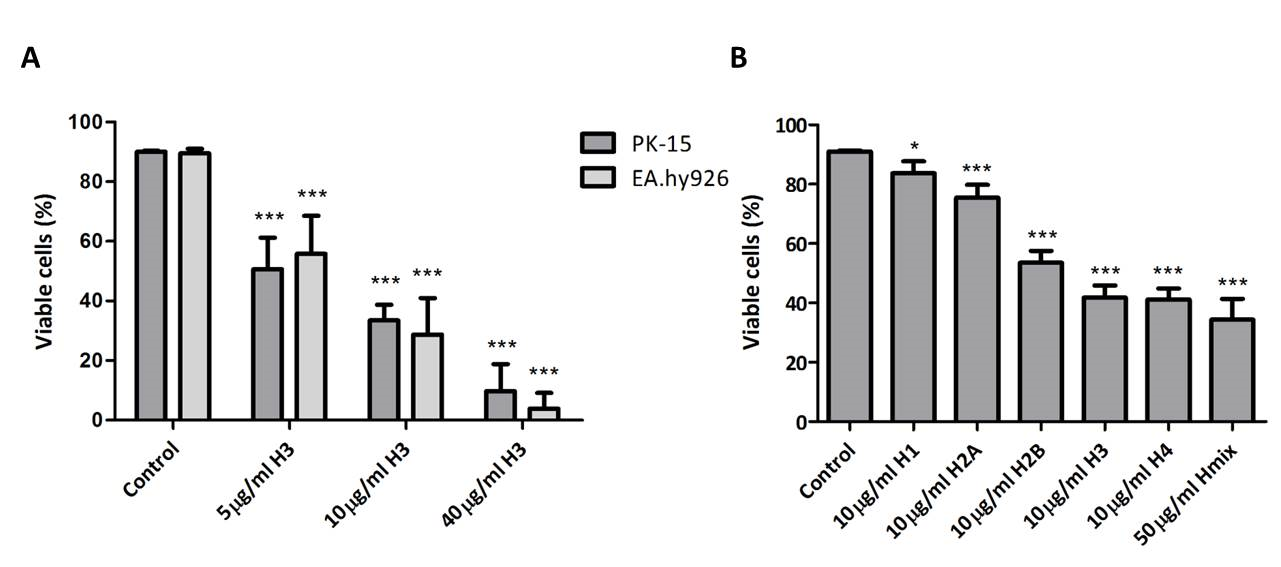

Supplement: S2 Fig — (A) Endothelial cells (EA.hy926) and porcine kidney epithelial cells (PK-15) were incubated with 5–40 μg/mL histone H3 in serum-free DMEM for 1 hour at 37°C. (B) PK-15 cells were incubated with 10 μg/mL of individual histones H1, H2A, H2B, H3 and H4, or a mixture of these histones (Hmix) under the same conditions as in (A). Cell viability was assessed using flow cytometry with PI and annexin-A5 FITC. Data is shown as the mean + SD. *p<0.05 and ***p<0.001 as compared to control. (TIF) [file pone.0279944.s002.tif]
