# Supplementary material for: Extracellular histone release by renal cells after warm and cold ischemic kidney injury: Studies in an ex-vivo porcine kidney perfusion model
Source: PLoS One. 2023 Jan 20;18(1):e0279944. doi: 10.1371/journal.pone.0279944 (PMC9858092; doi:10.1371/journal.pone.0279944)
Supplement: S1 Table — (PDF) [file pone.0279944.s003.pdf]

S1 Table: Friedman and Mann-Whitney tests for differences between type of perfusion group per time point

		H3 (5 time points)	Glucose	LDH	Lactate	GST	pH	pO2	pCO2
Friedman HMP	Chi-square	6.333	18.000	16.914	24.361	1.172	11.075	9.257	7.387
	p-value	0.176	.001*	.002*	<.001*	.883	.026*	.055	.117
Friedman SNMP	Chi-square	18.719	16.000	23.886	28.000	5.600	14.071	8.547	1.640
	p-value	.001*	.003*	<.001*	<.001*	.231	.007*	.073	.802
10 min	HMP median (IQR)	0.00 (0.00-0.00)	9.9 (9.8-10.3)	5 (4-11)	0.6 (0.3-0.7)	7.9 (3.5-9.9)	6.99 (6.96-7.08)	26.0 (24.6-27.6)	2.9 (2.3-3.8)
	SNMP median (IQR)	0.00 (0.00-0.00)	9.3 (9.1-9.9)	11 (8-23)	1.0 (0.4-1.0)	7.9 (6.4-10.2)	6.84 (6.82-6.87)	25.6 (24.4-26.1)	5.6 (4.9-5.6)
	Mann-Whitney p-value	1.000	.035*	.165	0.259	.818	.001*	.383	.017*
60 min	HMP median (IQR)	0.00 (0.00-0.00)	8.7 (8.4-9.2)	21 (17-25)	0.6 (0.6-0.8)	6.5 (4.1-11.7)	6.89 (6.81-6.97)	23.5 (21.7-24.9)	4.0 (2.8-6.9)
	SNMP median (IQR)	0.00 (0.00-0.10)	8.1 (7.7-8.3)	40 (32-47)	2.2 (1.5-2.7)	10.3 (7.3-12.2)	6.80 (6.80-6.84)	23.5 (20.9-25.3)	5.4 (4.6-9.9)
	Mann-Whitney p-value	.805	.015*	.001*	.001*	.366	.038*	.902	.128
120 min	HMP median (IQR)	0.00 (0.00-0.05)	8.7 (8.1-8.8)	36 (34-48)	0.8 (0.7-1.1)	10.0 (5.0-10.9)	6.89 (6.80-6.90)	24.8 (24.2-25.7)	4.3 (3.5-6.4)
	SNMP median (IQR)	0.15 (0.00-0.22)	7.5 (7.0-7.7)	60 (58-85)	4.0 (2.6-4.4)	11.4 (6.3-13.7)	6.80 (6.80-6.80)	23.7 (22.8-24.6)	7.9 (4.5-9.3)
	Mann-Whitney p-value	.097	.001*	.038*	.001*	.318	.038*	.128	.073
180 min	HMP median (IQR)	0.00 (0.00-0.00)	8.2 (7.6-8.6)	48 (35-51)	1.0 (0.7-1.0)	8.5 (8.4-10.2)	6.86 (6.80-7.00)	24.0 (22.6-26.0)	4.9 (2.9-7.0)
	SNMP median (IQR)	0.11 (0.02-0.26)	6.9 (6.2-7.2)	95 (76-117)	5.7 (3.6-6.4)	10.9 (6.6-12.8)	6.80 (6.80-6.80)	22.9 (21.1-24.9)	7.1 (4.8-7.7)
	Mann-Whitney p-value	.011*	.008*	.004*	.001*	.383	.026*	.456	.209
240 min	HMP median (IQR)	0.02 (0.00-0.06)	8.2 (8.1-8.5)	56 (41-62)	1.0 (0.9-1.1)	11.3 (5.5-12.6)	6.90 (6.80-6.94)	22.8 (22.0-24.7)	4.3 (2.8-8.6)
	SNMP median (IQR)	0.48 (0.20-0.83)	6.0 (5.7-7.1)	118 (61-130)	7.4 (5.2-9.0)	10.4 (7.5-13.2)	6.80 (6.80-6.81)	24.1 (23.1-25.8)	7.9 (5.6-8.7)
	Mann-Whitney p-value	.004*	.001*	.038*	.001*	.836	.165	.456	.165
